# Understanding the molecular basis of herbal medicines for cough variant asthma under the guidance of traditional herbal theories

**DOI:** 10.3389/fphar.2025.1594308

**Published:** 2025-06-11

**Authors:** Rui Liu, Jiaqi Yao, Yihang Sui, Yinnan Zhang, Boon Seng Kho, Yingli Zhu, Ninghua Tan, Yinyin Wang

**Affiliations:** ^1^ Department of TCMs Pharmaceuticals, School of Traditional Chinese Pharmacy, China Pharmaceutical University, Nanjing, China; ^2^ School of Chinese Materia Medica, Beijing University of Chinese Medicine, Beijing, China

**Keywords:** herbal medicine theory, graph neural networks, meridian theory, network proximity, suhuang formula, cough variant asthma (CVA)

## Abstract

**Introduction:**

Despite the clear clinical efficacy of the herbal formula *Suhuang* in treating cough variant asthma (CVA), its underlying mechanisms of action (MOAs) remain poorly understood. Traditional Chinese Medicine (TCM) offers a unique framework for disease treatment based on traditional herbal theories. However, the molecular basis of these theories remains largely unexplored.

**Methods:**

To address this gap, we proposed a novel computational paradigm to understand how herbal medicines exert therapeutic effects on CVA under the guidance of TCM theories. Our approach integrates transcriptional perturbation data, graph neural network (GNN) models, and network proximity analysis, enabling the interpretation of herbal actions within a network pharmacology context.

**Results:**

We found that traditional herbal theories show strong molecular-level associations with therapeutic mechanisms: 1) Meridian classifications of herbs align with their gene perturbation profiles across different organs; 2) Herbal combinations and their therapeutic efficacy correlate with the network proximity of their targets to disease-specific genes. Notably, network proximity analysis revealed mechanistic support for key TCM concepts such as the *JUN-CHEN-ZUO-SHI* hierarchy and the Lung‐Large Intestine Theory; 3) By incorporating features derived from traditional herbal theory, we developed two GNN-based models to predict herb‐disease associations and herb‐herb combinations, which identified potential active ingredients and synergistic formulations for CVA.

**Discussion:**

This study presents a novel framework for interpreting the molecular basis of herbal medicines and their combinations under TCM theory guidance.

## 1 Introduction

Cough Variant Asthma (CVA) is a respiratory condition characterized by bronchial hyperresponsiveness, airway inflammation, and remodeling, leading to severe and frequent coughs (Padem and Saltoun, 2019; Diab et al., 2022). CVA’s clinical manifestations include paroxysmal, spasmodic, and repetitive coughing, which align with the “wind cough” syndrome in herbal medicine theory. Herbal medicine has played a pivotal role in disease prevention and treatment for thousands of years. Moreover, herbal medicine and natural products are increasingly recognized globally as valuable resources for drug discovery. Herbal medicine employs a holistic approach, utilizing multiple ingredients, targets, and pathways to achieve therapeutic efficacy (Kim et al., 2015). For instance, the Suhuang formula for CVA and post-infectious cough (PIC) is considered to be associated with “wind cough” syndrome in herbal medicine theories. It consists of nine herbs and is primarily used to alleviate cough, throat itching, and related symptoms caused by “wind evil invading the lung.” While the clinical efficacy of Suhuang is remarkable, its underlying mechanism of action (MOA) in treating CVA remains to be fully elucidated (Qin et al., 2019; Guo et al., 2021; Jiang et al., 2023).

Unlike modern medicine, herbal medicine is under the guidance of particular theories, such as Four Qi (Energies), Five Flavors, Twelve Meridian Tropism, and 37 herbal syndromes in herbal medicine. Herbal medicine theories are the foundation and guidelines of disease treatment. Many herbs and their ingredients have been linked to various modern diseases, corresponding with their classification within herbal medicine theory. For example, the herb *Salvia miltiorrhiz*, associated with the heart meridian, has a long history of use in treating chest discomfort and heart pain. Recently, a compound extracted from *Salvia miltiorrhiza* named Tanshinone has been found to have cardioprotective effects in clinical settings for cardiovascular disease (Zhang et al., 2019; Xu et al., 2022), aligning with its traditional therapeutic role. Another example is the *Rheum palmatum* from the Liver Meridian, as demonstrated by the ingredient emodin extracted from *R. palmatum* that could alleviate liver fibrosis via Nur77-mediated epigenetic regulation of GLS1 (Sharma et al., 2022; Chen et al., 2023). Thus, it is meaningful to explore how traditional herbal theories guide the treatment of CVA with the Suhuang formula.

Recently, many novel computational pharmacology frameworks have been proposed by integrating multi-modal data for drug discovery and herbal medicine, such as MUSCLE (Ji et al., 2024b), scDCA (Ji et al., 2024a), CellMsg (Xia et al., 2024), and GraRep (Ji et al., 2020), deepening our understanding of complex diseases. These computational methodologies also show promise in exploring the essence of traditional herbal theories for disease treatment. For example, Yinyin Wang et al. developed a meridian prediction model using machine learning techniques, uncovering molecular mechanisms underlying these theories (Wang et al., 2019). Network-based methods have proven to be powerful tools for identifying intrinsic relationships within complex herbal formulations (Menche et al., 2015; Cheng et al., 2019). Our previous work demonstrated that network proximity algorithms could effectively capture the biological interactions between herbal ingredients (Wang et al., 2021). Similarly, Hu et al. revealed the molecular mechanisms of Fuzi Lizhong Pill and Huangqin Decoction in treating the cold and heat syndromes of ulcerative colitis through network pharmacology (Hu et al., 2023). Especially, graph neural network framework, such as GAT (Graph Attention Network) and SEAL (Subgraph-based Embedding for Attributed Link Prediction), are relatively complex graph neural network architectures that are well-suited for capturing complex relationships in graph-structured data. GAT is suitable for multi-label classification tasks, as its self-attention mechanism can effectively capture the complex dependencies between nodes in the graph. GAT’s adaptive weighting allows it to perform exceptionally well in modeling the relationships between labels, especially when labels are interdependent. SEAL is specifically designed for link prediction tasks, SEAL learns subgraph embeddings to capture local structural information between nodes, which is particularly effective for predicting potential node connections in the graph and can efficiently handle large-scale graph data while incorporating node attributes.

Notably, integrating high-throughput sequencing with systems biology has led to significant advancements in herbal medicine research (Zhu et al., 2022). For example, Wu et al. employed integrated multi-omics and network pharmacology methods to explore the biological basis of syndrome differentiation in coronary heart disease patients with two distinct syndromes (Wu et al., 2021). Additionally, Li et al. utilized lipidomic and transcriptomic integration to investigate the therapeutic effects of Saikosaponin A and D on non-alcoholic fatty liver disease (Li et al., 2021). Also, Zhang et al. conducted a network pharmacological analysis of herbal medicine metabolomic data to identify diagnostic markers in herbal medicine (Zhang et al., 2020).

However, many of these studies have not fully integrated herbal medicine theoretical perspectives with omics data, thus overlooking the unique characteristics of herbal medicines. In this study, we aimed to elucidate how herbal medicine exerts therapeutic effects on CVA disease under the guidance of herbal medicine theories by integrating transcriptional perturbations. Using the Suhuang antitussive capsule as a case study, we first explored the relationship between herbal medicine theory classifications and tissue-specific expression of signature genes derived from transcriptional perturbations. Using the Suhuang antitussive capsule for the disease CVA as an example, we first investigated the association between herbal medicine theory classification and the tissue expression of signature genes from the transcriptional perturbation. With these theories as features, we developed graph neural network models that enable accurate prediction of potential herb-disease association and herb-herb combination models. More importantly, to understand the mechanism of actions of these predicted results, we propose a novel network proximity method that could integrate signature genes and targets to understand their efficacy from the view of traditional herbal theories. Overall, our study offers new insights into the molecular basis of herbal medicines by reviewing herbal theories through computational methods.

## 2 Materials and methods

### 2.1 Collection of the tissue distribution of herbal signature genes and their meridians

Given that the signature genes of herbal perturbation and meridian classifications are crucial for understanding herbal disease treatment, we first investigated the relationships between the tissue distribution of herb gene expression and their meridian classifications and the associations between CVA disease and herbal meridians.

Firstly, we collected high-throughput GEO datasets for herbs from the HERB database (Table 1). Then, the transcriptional expression (noted as “nTPM”) on 54 different tissues of each gene was downloaded from the Human Protein Atlas, a comprehensive resource that maps human proteins across cells, tissues, and organs (Pontén et al., 2011). Extreme expression values greater than 1,000 were excluded. Then, we mapped relevant tissues to their corresponding ten meridians to explore the relationship between the genetic distribution landscape of herbal signature genes and the associated meridians.

**TABLE 1:** High-throughput GEO datasets for herbs collected in the HERB database.

Number	Herb name	HERB ID
1	Codonopsis pilosula	HERB001211
2	Rehmannia glutinosa	HERB001251
3	Ophiocordyceps sinensis	HERB001333
4	Astragalus membranaceus	HERB002560
5	Theobroma cacao	HERB003106
6	Ginkgo biloba	HERB000189
7	Gelsemium elegans	HERB000696
8	Solanum lycopersicum	HERB001616
9	Echinacea purpurea	HERB007181
10	Lepidium apetalum	HERB000523
11	Acetum	HERB000960
12	Glycine max	HERB001023
13	Allium sativum	HERB001104
14	Angelica sinensis	HERB001210
15	Glycine max oil	HERB001382
16	Propolis	HERB001705
17	Forsythia suspensa	HERB003361
18	Ganoderma lucidum	HERB003444
19	Camellia sinensis	HERB003617
20	Natrii Sulfas	HERB003748
21	Actinidia chinensis	HERB003790
22	Diospyros kaki	HERB005109
23	Agaricus bisporus	HERB005147
24	Linum usitatissimum	HERB006326
25	Cordyceps militaris	HERB006666
26	Cinnamomum camphora	HERB006931

In addition to individual herbs, we used the Suhuang formula for CVA as an example to further explore the relationship between the tissue distribution of the herbal signature genes from the CVA formula and the meridians of each herb within the formula. The signature genes of Suhuang in the CVA animal model, identified in our previous study, were used to represent gene perturbation (Wang et al., 2024).

### 2.2 Herb-disease association prediction using graph attention network (GAT) model

With herbal theories as critical features for disease treatment, we further developed an herb repurposing computational model to screen effective herbs for CVA on a larger scale. To prepare the training datasets, traditional Chinese herbal medicines and their 199 properties in herbal medicine theory were collected from TCMID databases and prepared as feature vectors by one-hot encoding method, including Four Qi (Energies), Five Flavors, Twelve Meridian Tropism, and 37 herbal syndromes. Moreover, to represent the herb-herb interaction graph, herb prescriptions were collected from the literature to calculate the co-existence between two herbs. Additionally, herb-disease associations were collected from SyMMap databases.

Feature Matrix Construction: The feature matrix of herb node was denoted as $X\in R^{n*f}$ , where n is the number of nodes and f is the number of features in graph X . Each row $X_{i}$ represents the feature vector for herb i , encompassing its characteristics such as Qi, Favors, Meridian Tropism, and syndromes.

Adjacency Matrix Construction: Correspondingly, based on the herbal pairs, an adjacency matrix $A\in R^{n*n}$ was constructed to represent the relationships between different herbs. $A_{i,j}$ = 1 if the co-existence frequency between node i and j is larger than 5 otherwise $A_{i,j}$ = 0.

Label Matrix Construction: A label matrix $Y\in R^{n*c}$ was generated based on the disease tags associated with each herb. c is the number of disease categories ( c=14 here) and n is the number of herb node. Each row $Y_{i}$ represents the disease category vector for herb i . The elements of $Y_{i}$ are binary, indicating whether herb is associated with a particular disease category. $Y_{i,j}$ = 1 if herb i is associated with disease category c otherwise $Y_{i,j}=0$ .

As shown in Figure 3A, the GAT model processes the feature matrix X and the adjacency matrix A to learn a representation for each herb that is useful for predicting its associated diseases. The GAT model was initialized with two graph attention layers. The forward propagation included two layers, each supplemented with a dropout layer to prevent overfitting. The hidden layers utilized Leaky ReLU activation functions, the output of the network was computed as shown in Equation 1.

$$H^{\left(l+1\right)}=\sigma \left(\sum_{j\in N\left(i\right)}\alpha_{ij}^{\left(l\right)}\;W^{\left(l\right)}H_{j}^{\left(l\right)}\right)$$ (1)

where $H^{\left(l\right)}$ is the matrix of activations at layer l , with $H^{\left(0\right)}=X$ ; $W^{\left(l\right)}$ is the weight matrix for layer l ; $\alpha_{ij}^{\left(l\right)}$ is the attention coefficient between nodes i and j , computed as a function of their features; $N\left(i\right)$ is the set of neighbors of node i in graph; σ is a non-linear activation function Leaky ReLU. The attention coefficients are computed using Equation 2:

$$\alpha_{ij}=\frac{\exp\left(\text{LeakyReLU}\left(a^{T}\left[Wh_{i}\left\|W\right.h_{i}\right]\right)\right)}{\sum_{k\in N\left(i\right)}\exp\left(\text{LeakyReLU}\left(a^{T}\left[Wh_{i}\left\|W\right.h_{k}\right]\right)\right)}$$ (2)

where a is a weight vector and “‖” denotes concatenation.

The GAT model was used with an input feature dimension of 199, a hidden layer dimension of 256, and an output dimension of 14 with binary cross-entropy loss as loss function. During training, we performed hyperparameter tuning to identify the best configuration for our model, including learning rate, hidden layer size, dropout rate, and attention heads. In detail, the tuning process included the following settings: 1) Learning Rate: A range of values between {0.001, 0.1} was tested using the Adam optimizer; 2) Hidden Layer Size: We selected hidden layer sizes from {64, 128, 256} to find the most effective architecture; 3) Dropout Rate: We experimented with dropout rates of {0.1, 0.2, 0.3, 0.4, 0.5, 0.6, 0.7} to prevent overfitting; 4) Attention Heads: To improve the model’s expressive power, eight attention heads were chosen for better information propagation. To prevent overfitting, we implemented early stopping with a patience value of 10 and trained the model for 100 epochs. The hyperparameters were selected through extensive experimentation to ensure the model’s stability and strong performance.

The model’s predictions were assessed using four metrics: average precision score (AP), roc_auc_score (ROC), label ranking average precision score (LRAP), and label ranking loss (LRL). Scores closer to “1” for the first three metrics indicate higher accuracy, while lower scores for label ranking loss indicate better performance. The dataset was divided into 60% training, 20% validation, and 20% test sets. After determining the optimal parameters for herb-disease association models, the novel herbs with high prediction score on CVA disease were prioritized and considered as potential herbs for CVA disease treatment.

### 2.3 Herb-herb combination models by graph neural network SEAL

Since herbal medicines are usually used in combination for optimal effects, we further developed herb-herb combination models to recommend potential synergistic herbs for CVA disease. Based on known herb co-occurrence in herbal prescriptions, all existing links between herb-herb from adjacency matrix A (value 1 in A) were selected as positive samples. The dataset was divided into training (60%), validation (20%), and testing (20%) sets. Similarly, an equal number of non-links (value 0 in A ) were chosen, with an identical split as negative samples.

For each positive link (existing connection) in the adjacency matrix A , a subgraph $G_{p}$ is extracted, and similarly for each negative link, a subgraph $G_{n}.$ For each node v in the subgraph G ( $G_{p}$ or $G_{n}$ ) is assigned a label $l_{v}$ . The node information matrix for a subgraph G is represented as $M_{G}=\left\{\left(l_{v},e_{v},f_{v}\right)\left|v\in \right.G\right\}.$ Embeddings $e_{v}$ are generated for node v to capture its structural and contextual properties within the global graph. It should be noted that embeddings $e_{v}$ from the pre-trained model. The features $f_{v}$ of each node v are derived from the original characteristics of the herbs, e.g., Qi, Flavors, Meridian Tropism, and syndromes. The SEAL model employs a Deep Graph Convolutional Neural Network (DGCNN) (Zhang et al., 2018) for processing the subgraphs. The GCN layer for a node v is given by Equation 3:

$$H_{v}^{\left(l+1\right)}=\sigma \left(W^{\left(l\right)}\sum_{u\in N\left(v\right)}\frac{1}{\sqrt{d_{v}d_{u}}}\;H_{u}^{\left(l\right)}\right)$$ (3)

where $H_{v}^{\left(l\right)}$ is the embedding of node v at layer l ; $W^{\left(l\right)}$ is the weight matrix for layer l ; $N\left(i\right)$ is the set of neighbors of node v in graph; $d_{v}$ and $d_{u}$ are the degree of node v and u ; σ is a non-linear activation function Leaky ReLU. The model is trained using the binary cross-entropy loss, as shown in Equation 4:

$$L=-\frac{1}{N}\sum_{i=1}^{N}\left[y_{i\;}.\log\left(\sigma \left(x_{i}\right)\right)+\left(1-y_{i\;}\right).\log\left(1-\sigma \left(x_{i}\right)\right)\right]$$ (4)

where N is the number of observations in the batch; $y_{i\;}$ is the true label for the i th observation (0 or 1); $x_{i\;}$ is the logit (raw output) of the model for the i th observation; $\sigma \left(x_{i}\right)$ is the sigmoid function applied to i th observation $x_{i\;}$ , which transforms the logit into a probability (between 0 and 1). The sigmoid function $\sigma \left(x\right)$ is defined as shown in Equation 5:

$$\sigma \left(x\right)=-\frac{1}{1+e^{-x}}$$ (5)

As shown in Figure 3A, the model consists of graph convolutional layer and global sort pooling layer to capture essential features from the graph data. These features are further processed through 1D convolutional layers and fully connected layers. To optimize the model’s performance, we conducted extensive hyperparameter tuning. The learning rate was searched in {0.0001, 0.001, 0.01, 0.1} with the Adam optimizer, and the loss function was selected as BCEWithLogitsLoss, given the binary classification nature of the task. To prevent overfitting, we experimented with different dropout rates and early stopping criteria, ultimately adopting a dropout rate of 0.5 and a patience of 10 for early stopping. Hyperparameters were optimized based on extensive experimental evaluation to enhance model stability.

### 2.4 Applying network proximity methods to understand the molecular MOAs of herbal medicine

To uncover the underlying mechanisms of herbal medicine’s therapeutic and synergistic effects, we propose a novel network proximity framework to explore the association between these theoretical properties and their role in disease treatment.

Using the Suhuang formula for CVA as an example, we compiled the ingredients of its nine herbs from six prominent herbal medicine databases, including Encyclopedia of Traditional Chinese Medicine (ETCM) (Xu et al., 2019), the Traditional Chinese Medicine Systems Pharmacology (TCMSP) (Ru et al., 2014), the Traditional Chinese Medicine Integrated Database (TCMID) (Zhang et al., 2022), the Traditional Chinese Medicine on Immuno-Oncology (TCMIO) database (Liu et al., 2020), the Traditional Chinese Medicine Information Database (TCM-ID) (Huang et al., 2018) and TCM-Mesh. Only those compounds existing in the PubChem database (Wang et al., 2009) were retained for further analysis. Moreover, ingredient-target relationships were extracted from the STITCH database (Szklarczyk et al., 2016), focusing on those with combined scores above 700. We also collected vital targets related to CVA with “cough variant asthma” as a keyword from the literature.

Then, we extracted protein-protein interaction (PPI) data from the STRING database (Szklarczyk et al., 2023) to construct a binary PPI network matrix, where “1” indicates a connection and “0” indicates no connection. This binary PPI network was then transformed into a Perturbation-Weighted PPI network (denoted as B ) by assigning edge weights based on the averaged log2FoldChange in gene expression between two connected targets (genes). These log2FoldChange values were derived from drug perturbation studies of Suhuang in CVA disease animal models.

We define the proximity distance between node i and node j in B as their shortest path length, as shown in Equation 6:

$$Distance\left(i,j\right)=\min\left(\left\{\sum_{p^{\prime }\in P}w\left(e\right):P\;the\;pathes\;from\;i\;to\;j\;in\;graph\;B\right\}\right)$$ (6)

Where P represents all potential paths from node i to node j in the graph B. $p^{\prime }$ is one of the paths with $w\left(p^{\prime }\right)$ as the summarized weight of all edges in this path.

Based on the Perturbation-Weighted PPI network B , we calculated the $Distance\left(i,j\right)$ for pairwise herb targets, disease genes, and signature genes, resulting in a Distance matrix $C\in R^{n_{signature\_genes}*n_{targets/disease\_genes\;}}$ , where $n_{signature\_genes}$ represent the number of Suhuang signature genes, and $n_{targets}$ or $n_{disease\_gene}$ as the number of targets for Suhuang or CVA disease-related genes. Only signature genes and ingredient targets/disease genes with the top 5% shortest distances were considered significant associations.

The distances of one target with all signature genes indicate the general contribution of this target to herbal perturbation at the gene expression level. We then performed statistical analysis on the distances of targets associated with different herbal theories by mapping the targets to ingredients and subsequently to the herbs.

## 3 Results

### 3.1 Overview of this study

This study utilized machine learning and network-based methods to explore herbal medical theories, focusing on the Suhuang antitussive capsule for CVA as a case study. Meridian theories are a unique aspect of traditional medicine that helps determine therapeutic effects on various diseases. Additionally, gene perturbation induced by herbal medication is referred to gene signatures, offering valuable insights into the holistic effects of specific herbs or ingredients.

**FIGURE 1:**
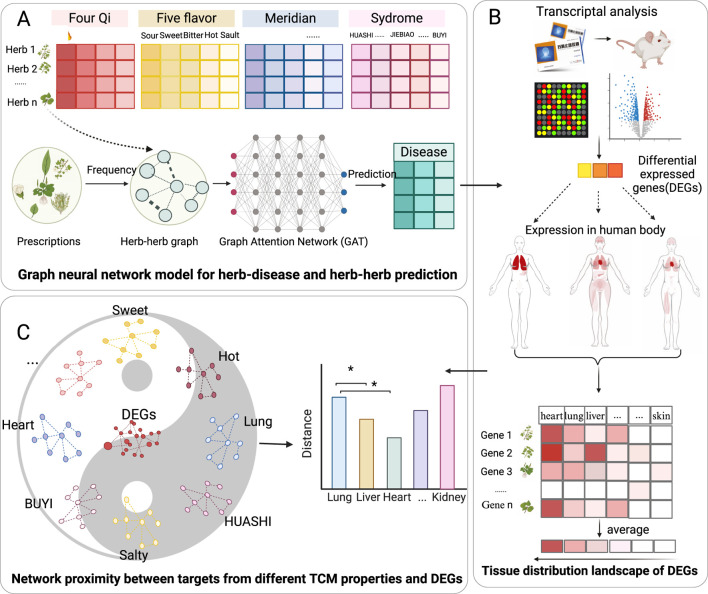
The overview of our study. **(A)** Interpretation of meridian theory by investigating the tissue distribution of differentially expressed genes (DEGs) of herbs on different tissue. **(B)** Developing graph neural network models to predict herb-disease associations and herb-herb combinations separately with herbal theories as features, including Four Qi, Five flavors, 12 meridians, and 37 herbal syndromes. The frequently co-existed herb-herb pairs from existing prescriptions were extracted as herb-herb linking graph. **(C)** Network proximity models for understanding the mechanism of action of predicted result from the view of herbal medicine theories.

**FIGURE 2:**
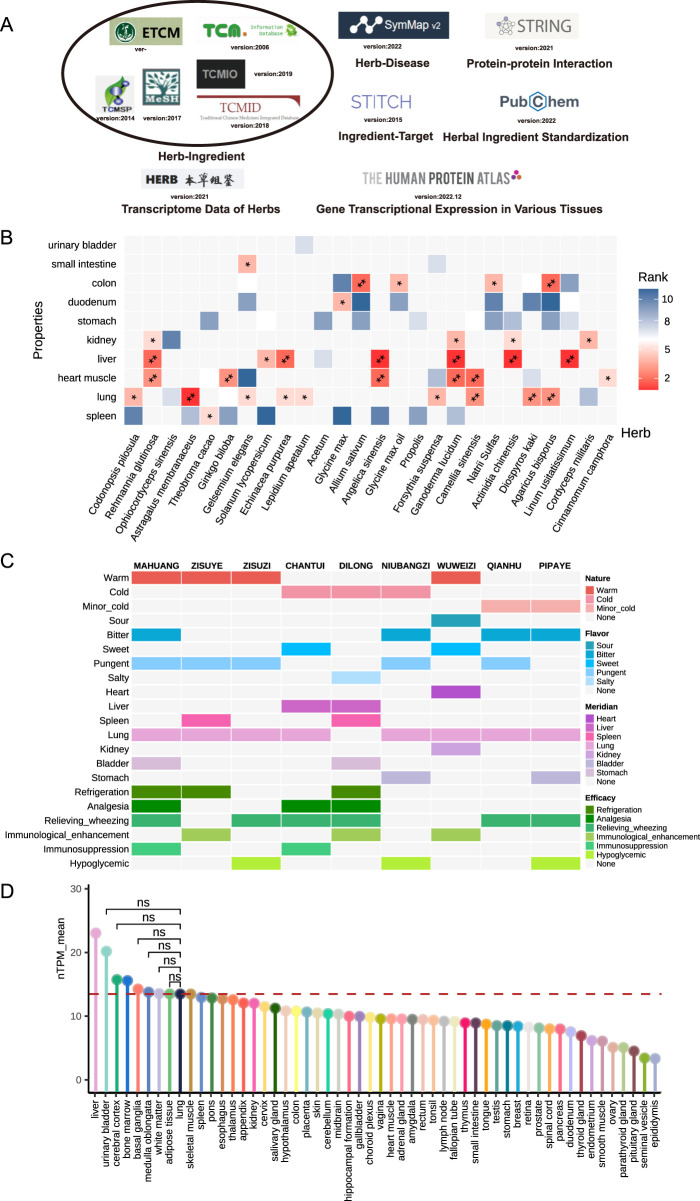
Systematic analysis on association between the tissue-specific expression and meridian across different herbs. **(A)** Data sources and database information used in this study. **(B)** Average expression of signature genes across 11 relevant tissues of 26 herbs with |Log_2_FoldChange|≥0.5 and *P-value* ≤0.05 as signature genes. **(C)** Theories distribution of herbal medicine in Suhuang formula with row as different herb and column as various theories. The colors in the heatmap likely denote the presence or intensity of these theories in each herb. **(D)** Expression of signature genes across nine herbs within Suhuang formula across 54 different tissues from the Human Protein Atlas databases.

However, the relationship between signature genes of herbal perturbations and herbal medicine theories remains unclear at the transcriptomic level. Therefore, in this study, we investigated the associations between CVA disease and herbal meridians from three main aspects (Figures 1A–C). Firstly, we explored how the signature perturbational gene of herbs from 12 meridians distributed in diverse tissues (Figure 1A). Secondly, we developed two graph neural network models for herb-disease association prediction and herb-herb combination prediction, incorporating traditional herbal theories as features, such as Four Qi, Five Flavors, 12 Meridians, and 37 herbal syndromes (Figure 1B). However, these prediction models lack interpretability of the underlying mechanisms of therapeutic and synergistic effects. Thus, we thirdly introduced a novel network proximity framework using the Suhuang formula for CVA disease as an example. This framework calculates the network distance between herb targets from different theories and signature genes, determining their significance in gene perturbation (Figure 1C). All databases utilized in this study and their versions were summarized in Figure 2A.

Through this multi-faceted analysis, our study aims to advance the molecular understanding of herbal theories in treating CVA disease and further facilitate drug discovery from natural products.

### 3.2 Systematic investigation on the correlation between tissue distribution of herbal signature genes and their meridians

To systematically evaluate the associations between the tissue distribution of herbal signature genes and their corresponding meridians, we collected transcript-level data for 26 herbs from the Herb database (Figure 2A). Signature genes for each herb were identified based on differential expression criteria of |log_2_FoldChange| ≥ 0.5 and *P-value* ≤ 0.05.

Our findings reveal that signature genes are predominantly expressed in tissues aligned with their associated meridians. As shown in Figure 2B, 23 out of the 26 herbs exhibited at least one meridian with significant expression in the corresponding tissues, highlighting a strong correlation between the tissue distribution of herbal signature genes and their herbal meridians. These results underscore the robust scientific significance of meridian theories, which have been developed over thousands of years, and suggest that they can be effectively deciphered at molecular and multi-omics levels. Therefore, incorporating traditional theories is essential when investigating herbal medicine disease treatments.

### 3.3 Understand the treatment effects of suhuang for CVA disease through tissue distribution of herbal signature genes and meridians

In addition to individual herbs, we investigated the Suhuang formula for CVA to explore the underlying relationships. Our previous research identified 589 signature genes (adjusted *P-value* < 0.05 and |log_2_FoldChange| > 2) associated with CVA treatment with Suhuang. The nine herbs in the Suhuang formula are mainly distributed in three natures, five flavors, seven meridian classifications, and six primary efficacies (Figure 2C).

To determine if the tissue distribution of these signature genes aligns with the meridian classifications of Suhuang, we examined their expression across various tissues. We observed that Lung tissue was among the top ten tissues with significantly higher gene expression compared to the other 43 tissues (Figure 2D). This finding corresponds with the characteristics of CVA disease, which is associated with respiratory functions and the Lung organ. Additionally, signature genes of the herbs in Suhuang exhibited high expression in Liver and Urinary Bladder tissues. This pattern is consistent with the herbal meridian theories: CHANTUI and DILONG correspond to the Liver meridian, while MAHUANG and DILONG align with the Bladder meridian (Figure 2C).

These results demonstrate that the gene expression patterns induced by Suhuang align with the meridian classifications of the herbs within the formula, suggesting a strong association between CVA and Lung tissue.

### 3.4 Herb-disease association prediction models with herbal theory properties as features

Having established the association between herbal properties and disease treatment, we developed graph neural network models to predict herb-disease associations and identify effective herbs for CVA disease, incorporating herbal medicine properties as features.

**FIGURE 3:**
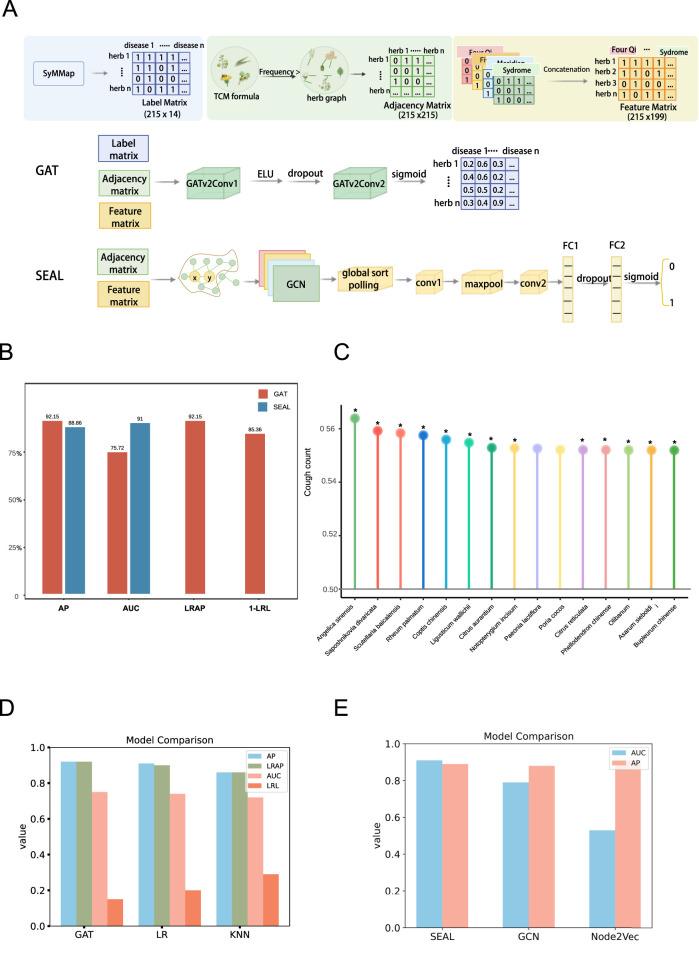
Performance of herb-disease association and herb-herb combination prediction models. **(A)** Workflow of the GAT model and the SEAL model. **(B)** The performance of Graph Attention Networks (GAT) model for herb-disease prediction and the SEAL model for herb-herb combination prediction across five key metrics. **(C)** The top 15 herbs with highest probability for CVA disease by herb-disease association model. The star sign (*) indicates that the therapeutic effect has confirmed by relevant experimental evidence or medication record. **(D)** Comparison of the GAT model with LR and KNN models based on AP, AUC, LRAP, and LRL evaluation metrics. **(E)** Comparison of the SEAL model with GCN and Node2vec models based on AP and AUC evaluation metrics.

We collected information on 7,518 herbs and their 177 theoretical properties from the TCMID database (Figure 2A). To accurately represent herb-herb interactions, we extracted 14,544 herbal pairs from 46,929 herbal prescriptions, retaining only those pairs that appeared in more than five prescriptions. This resulted in 3,691 significant herb-herb interaction pairs. Additionally, we gathered 149,805 herb-disease associations from the SyMMap database, covering 365 traditional Chinese herbs. We selected 221 common herbs for the graph-based model development, integrating herbal features (graph node features), herb-herb pairs (graph edges), and herb-disease associations (graph prediction labels) (Figure 3A).

After optimizing hyperparameters, we identified the best-performing models. The GAT model for herb-disease prediction achieved high accuracy, with an AP of 92.15%, LRAP of 92.15%, AUC of 75.72%, and a LRL of 14.64% Figure 3B. In addition, we compared the GAT model with logistic regression (Cox, 1958) and KNN (Cover and Hart, 1967) models, as shown in Figure 3D. In terms of the AP, GAT achieved a 1.15% improvement. For the LRAP, GAT showed a 2% increase. Regarding the AUC, GAT improved by 1%. Meanwhile, in terms of the LRL, GAT exhibited a 5% reduction.

The robust performance of these models highlights the significant role of traditional herbal theories in understanding and leveraging herbal medicines. These models enhance the ability to explore novel herbs for specific diseases, potentially expanding opportunities for drug repositioning.

### 3.5 Prioritization of potential herbs for CVA treatment using the herb-disease prediction model

We further utilized the herb-disease association model to identify potential herbs for effective CVA treatment. The framework of the SEAL model for herb-herb combination was depicted in Figure 3A. 211 herbs were scored to indicate its likelihood of being associated with CVA disease.

The top 15 herbs with the highest probability scores are presented in Figure 3C. Notably, 13 of these top herbs have been experimentally reported to have relevant therapeutic effects on CVA-related symptoms (Yingshu, 2010; Li, 2015; Ming-lei and Zhen, 2019; Amanguli, 2022; DU et al., 2022; Liang, 2022; Wang et al., 2023; Yue, 2023; Liu et al., 2024). For instance, *Angelica sinensis* was reported to significantly extend cough latency in mice and reduce cough frequency through ferulic acid (DU et al., 2022). Similarly, *Saposhnikovia divaricates*, and *Scutellaria baicalensis* are commonly used for treating coughs in herbal medicine, with the effects of dispelling wind, relieving colds, and possessing antibacterial and antiviral properties (Yingshu, 2010). On the other hand, the main components of *S. baicalensis* contain Baicalin and Baicalein, which exhibit anti-inflammatory, anti-allergic, and cough-suppressing effects (Liang, 2022), helping to reduce inflammatory responses and coughing symptoms effectively. These results demonstrate that our model is promising for discovering novel herbs for disease treatment. The remaining high-scoring herbs also warrant further validation to assess their potential efficacy in CVA treatment.

### 3.6 Recommendation models for the herb-herb combination using herbal theory properties as features

A key characteristic of herbal medicines is the combination of multiple herbs to achieve optimal therapeutic effects, guided by herbal medicine theories. We developed a SEAL recommendation model based on known herb co-existence in prescriptions for herb-herb combinations to predict potential synergistic herb pairs for disease CVA.

This model constructed an herb-herb graph by utilizing 3,691 herb-herb pairs extracted from herbal medicine prescriptions. SEAL is an advanced link prediction framework that represents each target link by extracting a local subgraph around it. The SEAL model demonstrated superior performance in terms of precision, accuracy, and lower prediction error. Specifically, the model achieved an AP score of 88.86% and an AUC score of 91.00% (Figure 3B).

To further evaluate the effectiveness of SEAL model, we compared its performance against several baseline models, including GCN (Kipf and Welling, 2016) and Node2vec (Grover and Leskovec, 2016). As shown in Figure 3E, SEAL consistently outperformed the baseline methods across multiple evaluation metrics. These results not only validate the effectiveness of SEAL, but also highlight the broader implications of herb-herb prediction. The high accuracy in predicting herb-herb combinations underscores the crucial importance of traditional prescriptions in discovering novel combination strategies for disease treatment.

### 3.7 Understand the combinational mechanism of suhuang through integrative analysis of signature genes and targets via network proximity methods

Despite the accurate performance of herb-disease and herb-herb combination association models, these models are not interpretable. To uncover the underlying mechanisms of therapeutic and synergistic effects, we propose a novel network proximity framework to understand the molecular basis of disease treatment from a view of the theoretical properties. Our hypothesis is that network proximity between the targets and signature genes of the Suhuang formula can represent their interactions, thereby helping to elucidate the mechanisms behind the herbal combinational theory.

**FIGURE 4:**
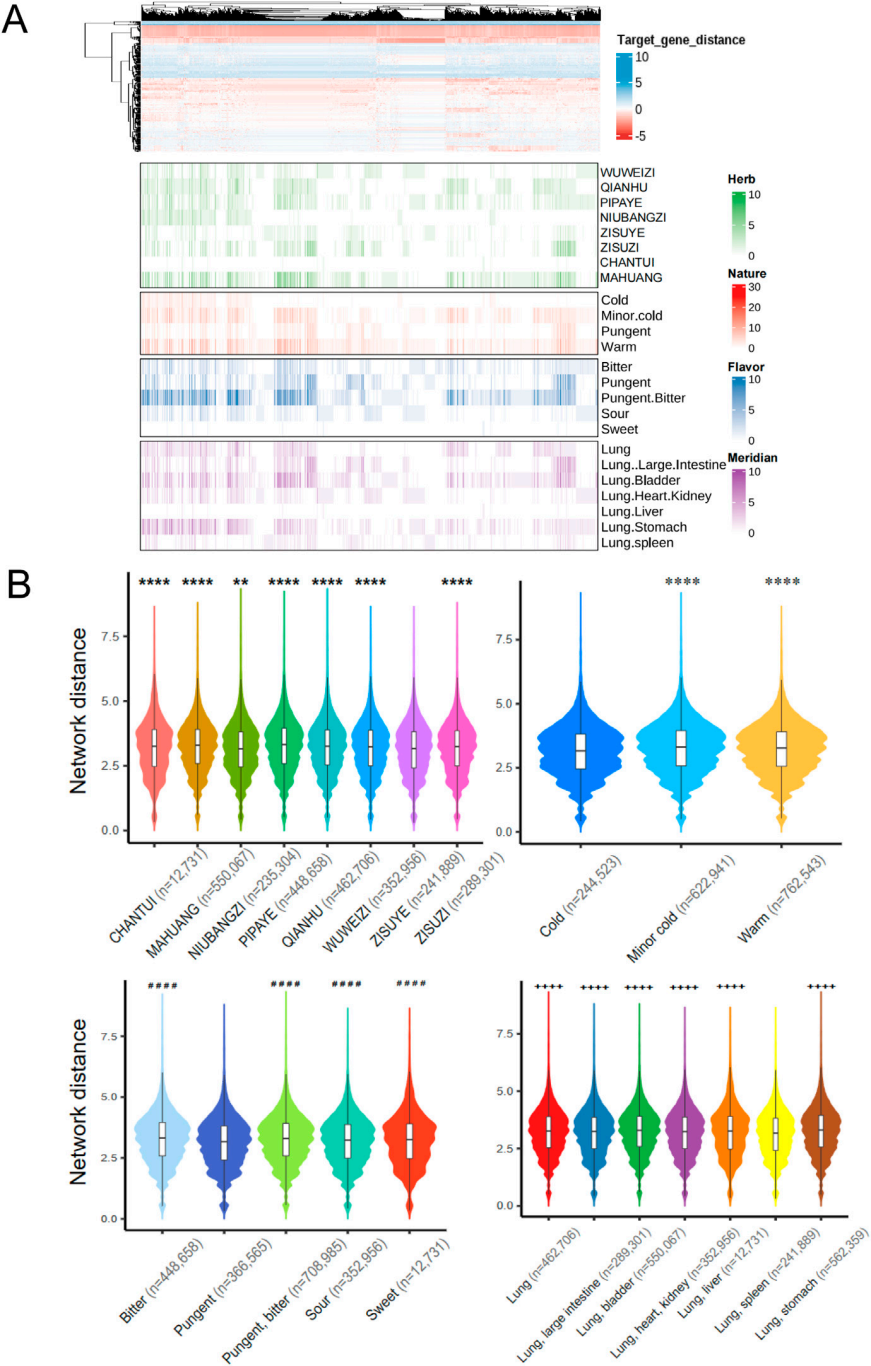
Integrated analysis of mechanism of action of Suhuang formula by network distance between targets and herbal theories. **(A)** The distribution of network distance between the signature genes and targets across different herbs in Suhuang as well as their corresponding herbal therapies. **(B)** Comparison of the network distance across different herbs, four natures, five flavor, and meridian. *P-values* were calculated by t.test. **p < 0.01, ****p < 0.0001 vs. the ZISUYE herb; ****p < 0.0001 vs. the Cold nature; ^####^ *p* < 0.0001 vs. the Pungent flavor; ^++++^ *p* < 0.0001 vs. the Lung, spleen meridian. **(C)** The comparison of the number of significant signature genes by fisher exact test in network C with specific theory as one group and other ones as another groups.

Using the Suhuang formula as a case study, we compiled data on 1,377 ingredients from six herbal medicine databases and 11,698 ingredient-target relationships from the STITCH database, covering 245 ingredients and 2,409 targets (Figure 2A). As shown in Figure 4A, the shortest path lengths between targets and signature gene interactions varied considerably, ranging from 0 to 1.29, highlighting the heterogeneity of these interactions across targets from different herbs.

We further analyzed the contributions of the nine herbs in the Suhuang formula by examining the network proximity of their targets with signature genes. Notably, targets associated with ZISUYE exhibited significantly shorter path lengths compared to other herbs (Figure 4B). This observation was confirmed by Fisher’s exact test on the number of significant target-gene interactions in network C (*P-value* = 3.56e^−64^) (Figure 4C), suggesting ZISUYE may cause significant drug perturbation in CVA disease animal models. This suggests that ZISUYE may induce significant drug perturbations in CVA disease models, aligning with its role as a principal (“JUN”) medicine in the Suhuang formula.

Comparing network proximities of targets and signature genes from different herbal theories revealed that targets from herbs with cold nature, pungent flavor, and Lung and Spleen meridians tended to exhibit significantly lower proximities, as well as a greater number of significant target-gene interactions in network C (Fisher’s exact test, *P-value* < 10^–4^) (Figure 4C). Additionally, targets from herbs associated with the Lung and Large Intestine meridians showed notably lower proximity (Figure 4B), reinforcing the relevance of these meridians in the context of Suhuang. The critical role of the Lung meridian in Suhuang’s therapeutic action is consistent with our findings regarding the tissue distribution patterns of signature genes in Lung tissue (Figure 2D). This finding also aligns with the “Lung-Large Intestine connection” in herbal medicine theories (Liu et al., 2012; He et al., 2021), which corresponds to the “gut-brain axis” in modern medicine (Agirman et al., 2021). Our previous research indicated that Suhuang may alleviate airway inflammation and sputum obstruction by promoting Hepatocyte Growth Factor (HGF) secretion through an intestinal endocrine-dependent pathway (Tong et al., 2019).

In summary, we propose a novel network-based framework that integrates target and signature gene information to better understand the synergistic mechanisms of herbal formulas from the perspective of traditional herbal therapies.

## 4 Discussion

Herbal medicines, particularly those from herbal medicine, represent a valuable resource for novel drug discovery. However, the significance of traditional herbal theories is often overlooked in modern scientific research. To address this gap, we propose an innovative approach that integrates traditional herbal theories with molecular understanding.

In this study, we emphasized the crucial role of traditional theories in our understanding of herbal disease treatment. Traditional theories are fundamental for computational modeling of herbal remedies, as they provide rich phenotype-based information, including herb-disease associations and herb-herb combination recommendations. These theoretical aspects of herbal medicine correspond to specific physiological states and treatment effects, which can be seen as processes aimed at restoring the body’s balance. For example, the meridian system in herbal medicine, which maps to biological pathways through Qi flows, is linked to various organs and bodily functions. Understanding which meridians are affected can help predict or diagnose which diseases a particular herbal remedy might address. However, due to limitations in available gene perturbation data, our study focused on the gene signatures of only 26 herbs to explore the scientific significance of traditional herbal theories. We anticipate that further research will expand upon these findings as additional drug perturbation datasets for more herbs become available.

Compared to other traditional machine learning methods, GNNs are particularly suited for modeling the complex interactions among herbal medicines. In this study, we developed advanced GNN models to accurately predict potential herb-disease and herb-herb combinations. For herbal medicines with multiple ingredients, GNNs can represent herbs and their properties as nodes, with interactions as edges, thereby uncovering underlying relationships. We believe our herb-disease association and herb-herb models are significant for discovering novel therapeutic strategies.

This study provides a unique perspective on CVA disease treatment by incorporating traditional herbal theories. Using the Suhuang formula as an example, we analyzed the tissue distribution of its signature genes in CVA animal models. Interestingly, we found that the tissue expression distribution of these genes aligns with Suhuang’s meridian classification, highlighting the role of the Lung meridian in CVA disease. In herbal medicine, the Lung meridian is linked with respiratory functions. This alignment between tissue expression and meridian classification reflects the potential convergence of traditional herbal medicine concepts with modern molecular biology.

Network proximity methods are particularly effective for understanding herbal combinations due to their ability to analyze complex interactions and align with holistic characteristics. Herbal medicine emphasizes a holistic approach, focusing on restoring balance within the body’s systems. Network proximity methods support this holistic view by analyzing entire networks of interactions rather than isolating individual components. Our results offer new insights into the molecular basis of these properties. For instance, ZISUYE, as a key herb in Suhuang, exhibited interaction patterns confirming its primary role in the formulation. Our study also supports the “JUN-CHEN-ZUO-SHI” theory in traditional medicine and underscores the importance of understanding herbal medicine properties in drug discovery. Current studies have confirmed that *Perilla frutescens* leaf extract can significantly attenuate LPS-induced pathological changes in lung tissue and improve respiratory function (Guo et al., 2025). These results suggest the biologically interpretable significance of our prioritized herb-disease and herb-herb associations.

While the findings of our study are promising, there are several limitations that should be considered. As our study relies on existing GEO datasets and databases, only 26 herbs with gene signatures were collected to explore the scientific significance of traditional herbal theories, which may introduce potential biases. This may affect the robustness and generalizability of our conclusions. We anticipate that future research, leveraging more comprehensive and diverse datasets, will help expand upon these initial findings, providing a clearer and more refined understanding of the molecular mechanisms involved.

## 5 Conclusion

In summary, this study integrates graph neural networks, gene perturbation data, and network-based methods to explore the principles of herbal medicine for CVA treatment. By grounding GNN models in traditional herbal theories, we accurately predicted herb-disease associations and herb-herb interactions. We also introduced a network proximity model to clarify the mechanisms behind these predictions. Our findings offer a robust framework for exploring the molecular foundation of herbal medicines and their combinations, showing how traditional theories can inform modern computational and molecular approaches to drug discovery.

## Data Availability

The original contributions presented in the study are included in the article/supplementary material, further inquiries can be directed to the corresponding authors.

## Abbreviations

CVA, cough variant asthma; GNN, graph neural network; PIC, post-infectious cough; GAT, graph attention network; DGCNN, Deep Graph Convolutional Neural Network; ETCM, Encyclopedia of Traditional Chinese Medicine; TCMSP, Traditional Chinese Medicine Systems Pharmacology; TCMID, Traditional Chinese Medicine Integrated Database; TCMIO, Traditional Chinese Medicine on Immuno-Oncology; TCM-ID, Traditional Chinese Medicine Information Database; PPI, protein-protein interaction; HGF, Hepatocyte Growth Factor.
